# Genomic Analyses of Major SARS-CoV-2 Variants Predicting Multiple Regions of Pathogenic and Transmissive Importance

**DOI:** 10.3390/v16020276

**Published:** 2024-02-10

**Authors:** Steven W. Brugger, Julianne H. Grose, Craig H. Decker, Brett E. Pickett, Mary F. Davis

**Affiliations:** Department of Microbiology and Molecular Biology, Brigham Young University, Provo, UT 84602, USAjulianne_grose@byu.edu (J.H.G.); brett_pickett@byu.edu (B.E.P.)

**Keywords:** SARS-CoV-2, genomics, bioinformatics, coevolution, codon bias

## Abstract

The rapid evolution of SARS-CoV-2 has fueled its global proliferation since its discovery in 2019, with several notable variants having been responsible for increases in cases of coronavirus disease 2019 (COVID-19). Analyses of codon bias and usage in these variants between phylogenetic clades or lineages may grant insights into the evolution of SARS-CoV-2 and identify target codons indicative of evolutionary or mutative trends that may prove useful in tracking or defending oneself against emerging strains. We processed a cohort of 120 SARS-CoV-2 genome sequences through a statistical and bioinformatic pipeline to identify codons presenting evidence of selective pressure as well as codon coevolution. We report the identification of two codon sites in the *orf8* and *N* genes demonstrating such evidence with real-world impacts on pathogenicity and transmissivity.

## 1. Introduction

SARS-CoV-2 is a rapidly evolving coronavirus first identified in Wuhan, China, in December of 2019 and has since spread extensively to create a global pandemic. Since the identification of its original strain, it has undergone a rapid evolution, resulting in several novel variants of notoriety, including Pango lineage B.1.1.7 (alpha), B.1.351 (beta), B.1.617.2 (delta), and B.1.1.529 (omicron), each with slightly altered levels of transmissivity and symptom severity. Due to how pervasively the virus has spread, together with the general increase in transmissivity with each successive prominent variant, experts worldwide have posited that coronavirus disease 2019 (COVID-19) may become as common as the flu and require regular updates to the currently available vaccines [1]. Thus, understanding the evolutionary trajectory of SARS-CoV-2 is pivotal to the continued worldwide management of coronavirus disease 2019 (COVID-19).

Current SARS-CoV-2 research heavily emphasizes genetic studies from a variety of perspectives. A core concept in the field of genetics is codon usage. SARS-CoV-2 has a positive-sense single-stranded RNA genome, which is used to produce the viral proteins needed for replication, packaging, and modulating intracellular processes of the host organism. The cellular machinery that translates RNA into protein polypeptides does so by reading the RNA in three-nucleotide sections, referred to as codons. In humans, these polypeptides make use of 20 different amino acids and are encoded by 61 unique codons with 3 additional codons acting as the termination signals. This redundancy allows for variation, with a given amino acid being coded by anywhere from one to six unique codons. The end result is that even homologous proteins composed of identical amino acid sequences may be encoded differently, at the nucleotide level, between pathogens in the same species through the use of different codons [2]. It has been reported that individual organisms possess biases for specific codons and that unique biases may even exist between organ systems within the same organism [2]. 

Several competing theories have been offered as to the origins of codon bias. The first posits that mutation is largely non-random and that certain nucleotides possess inherently higher mutation rates than others. Transition mutations (e.g., A < > G or C < > T) are generally more common than transversion mutations (A < > C, A < > T, G < > C, G < > T) [3]. This naturally leads to disproportionate codon mutation rates [3]. Another theory suggests that certain codons are selected over others in order to achieve increasingly efficient or accurate translations [4], as improved translational accuracy aids the organism in conserving precious cellular resources by preventing non-functional proteins from being inadvertently produced [3]. Supporting this theory is the finding that stronger codon biases have been observed in longer genes, likely due to the relatively higher resource cost to the organism for the mistranslation of large and/or critical proteins [5]. Further, codon bias may result from a differential natural abundance of tRNAs in an organism, as optimizing codon usage to match the levels of tRNA concentrations may be an effective strategy to balance supply and demand [6]. Genes have been observed to possess locally biased distributions of rare and frequent codons [7]. In addition, pauses during polypeptide synthesis are associated with the appearance of rarely used codons, while translation rates are nearly two times faster for polypeptides which employ more common codons [2]. It has been suggested that this may serve both to regulate the distribution of translating ribosomes across the mRNA, tune the protein’s co-translational folding processes, and facilitate protein translocation across membranes [8]. Finally, it has been noted that the identity of a codon’s third nucleotide exerts an influence on human mRNA stability, with G/C nucleotides conferring stability and A/T nucleotides conferring instability to the RNA strand [9], implicating some influence on gene expression overall. 

In reality, the factors underscoring codon biases are likely a combination of these theories, in conjunction with other external conditions, which define the codons most likely to aid the survival and reproduction of the organism. The existence and influence of codon bias, however, is not debated. No known organism has been observed to possess a full set of tRNAs with anticodons complementary to all 61 codons; humans, for example, possess only 45 tRNAs [3], with their relative abundance and presence varying widely among different tissues in a single organism [3]. It has been suggested that mutation bias may serve as an “orienting factor” in evolution, potentially influencing the predictability of a given trait arising by making some mutational trajectories more likely than others [10]. Regarding codon bias and its relationship with host–pathogen evolution, it has been demonstrated recently that some viruses that infect humans possess codon usage biases that align with the biases of the most highly expressed proteins in the tissues they infect [11]. This implies that, over time, some viruses may evolve to take advantage of the host’s cellular resources, likely including the most abundant tRNAs, resulting in a reproductive advantage and enhanced transmissivity. 

Given the multiplicity of factors governing viral evolution, including evidence that viral codon bias is incentivized to match that of the host tissue, we hypothesize that SARS-CoV-2 is experiencing a similar mutative pressure to align its codon bias with that of its host, resulting in a reproductive advantage and an increase in its transmissivity. If this hypothesis proves true, this knowledge will facilitate advanced preparations in the prevention and treatment of emerging SARS-CoV-2 strains.

## 2. Materials and Methods

### 2.1. Data Sources

The SARS-CoV-2 genomes were sourced from the GISAID database [12], which currently hosts over 13 million sequenced SARS-CoV-2 genomes. The variants for analysis were selected based on their current or previous classification by the Centers for Disease Control and Prevention as a “Variant of Concern” or “Variant of Interest”. The included variants consisted of the SARS-CoV-2 reference sequence and Pango phylogenetic lineages B.1.1.7 (alpha), B.1.351 (beta), B.1.429 (epsilon), B.1.525 (eta), B.1.526 (iota), B.1.1.529 (omicron), B.1.617.1 (kappa), B.1.617.2 (delta), P.1 (gamma), and P.2 (zeta). The candidate genomes were filtered based on the GISAID designation of completeness of the genome (>29,000 nucleotides in length with <1% N content) and a documented sample collection date. Genomes with a low coverage (>5% N content) were also excluded. Where possible, the samples included the first recorded instance of a new variant based on the reported GISAID accession numbers. The remaining samples were obtained by randomly selecting the genomes collected roughly within the first week of the identification of a new variant. The GISAID accession numbers of all the samples may be found in the Supplementary Materials (see Table S1). In addition to SARS-CoV-2 samples, we obtained a phylogenetic tree of all the included variants from GISAID for use in our downstream analyses. Subsets of the available sequences were used in place of all the potentially qualifying sequences of a variant due to limitations regarding mass accession of GISAID data with custom filters in place.

### 2.2. Data Preparation

The downstream analyses required that the input files contain only coding sequences with no stop codons present. Given that the process of translating genes orf1a and orf1b involves a frameshift approximately halfway through the orf1ab joint reading frame of the reference sequence, software packages designed to extract open reading frames failed to accurately capture the coding sequences for these genes. To remedy this, a custom Python script was written to automate the extraction of all 12 SARS-CoV-2 gene products. Input fasta files were searched for the start codon and subsequent three codons of each gene (as determined by the reference sequence) and parsed through the open reading frame until the first stop codon was located. The stop codon was removed from each gene sequence to ensure downstream compatibility with the analytical pipeline. Each SARS-CoV-2 gene was given its own multi-sequence fasta file containing the processed coding sequence for each gene product from each genome, for a total of 12 files from each genome. A multiple sequence alignment was then conducted on each fasta file using KCAlign, as it accounts for reading frames when creating and extending gaps in the generated alignment. 

### 2.3. Statistical Analysis

#### 2.3.1. Meta-CATS

The 12 processed multi-sequence fasta files from each genome served as the input for our analysis, and each gene was analyzed independently due to limitations in the analytical software used. To establish a baseline, codon biases for the human genome (HG) and human pulmonary tissue (HPT) were calculated based on established usage values [13], while bias in the SARS-CoV-2 genomes was calculated using the GISAID samples and averaged according to the variant. The codons labeled as “preferred” for a given amino acid were (1) the codons with the greatest percentage of usage, or (2) the codons whose usage fell within 10% of the top codon. Amino acids were permitted a maximum of two preferred codons per genome classification. In order to evaluate whether an identified variation in the provided genomes was significantly associated with any branches of the phylogenetic tree, a consensus variant analysis was executed using meta-CATS [14], which performs (1) a χ^2^ test of independence and (2) a Pearson’s χ^2^ test. As meta-CATS allows for comparisons between a maximum of 10 branches, Pango lineages B.1.617.1 and B.1.617.2 were grouped together for a total of 20 samples. In the cases of lineages B.1.525 and P.1, 10 samples from both Nigeria and the United Kingdom and from Brazil and Japan, respectively, were both included, as these variants emerged in two nations simultaneously. All the remaining samples were grouped by their respective Pango lineages. 

#### 2.3.2. HyPhy

Following the consensus variant analysis, a selective pressure analysis was conducted using the Fixed Effects Likelihood (FEL) method in HyPhy [15] to probe for evidence of a pervasive positive (i.e., diversifying) or negative (i.e., purifying) selection. The standard settings were used, and the input data remained the same as the preceding analysis, with the addition of the phylogenetic tree obtained from GISAID. Stop codons were removed from the fasta file for each gene, which was mandated by HyPhy.

#### 2.3.3. MISTIC

Finally, to examine whether the samples presented evidence of coevolving protein residues, the translated sequences were processed through the Mutual Information Server to Infer Coevolution platform (MISTIC) [16]. In this case, mutual information reflects the degree to which residue identity at a given position enables the prediction of other residue identities in a protein sequence and predicts which residues may be critically interdependent for protein function. These analyses were executed to characterize the impact of mutations that reinforce or depart from SARS-CoV-2’s existing bias on viral efficacy.

## 3. Results

**Figure 1:**
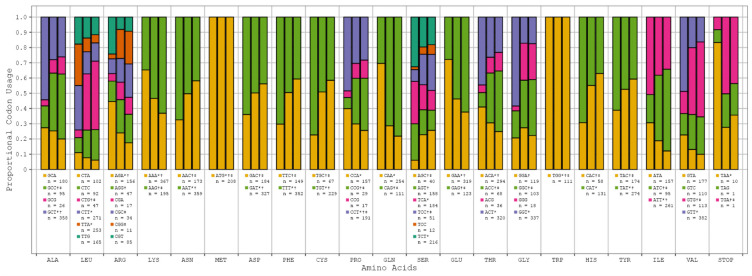
SARS-CoV-2 reference sequence vs. human genome vs. human pulmonary tissue codon bias. All the relevant amino acids with their respective codons and proportional usage across SARS-CoV-2 (*), HG (†), and HPT (‡) are displayed here. The columns in each amino acid grouping map to the SARS-CoV-2, HG, and HPT biases. The counts (n) below each codon are indicative of the total number of codons observed across all SARS-CoV-2 genome samples.

**Figure 2:**
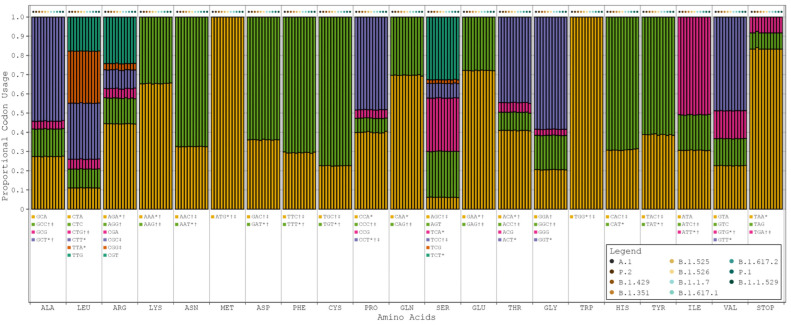
Proportional codon usage for SARS-CoV-2 reference sequence and major variants. Codon usage is calculated in terms of proportional usage per amino acid for each sample and averaged by variant. The order of bars in each grouping is as follows: A.1 (reference sequence), P.2 (zeta), B.1.429 (epsilon), B.1.351 (beta), B.1.525 (eta), B.1.526 (iota), B.1.1.7 (alpha), B.1.617.1 (kappa), B.1.617.2 (delta), P.1 (gamma), and B.1.1.529 (omicron). SARS-CoV-2 codon bias = *, HG codon bias = †, and HPT codon bias = ‡.

Progressive respiratory failure is the primary cause of death from SARS-CoV-2 infection, making human pulmonary tissue (HPT) codon bias of great interest [17]. We observed that the human genome (HG) and (HPT) codon biases were comparable, while the SARS-CoV-2 codon bias deviated from these substantially (see Figure 1, Table S2). However, among all the variants of SARS-CoV-2, codon bias was entirely consistent with only minor discrepancies in the precise proportions being noted. This suggests that the SARS-CoV-2 genome likely evolved independently of the translation machinery of the human host (see Figure 2). 

To determine whether any nucleotide substitutions played a role in the evolution of SARS-CoV-2 variant groups, we used the meta-CATS algorithm to identify sequence positions that had a statistically significant skew between two or more groups of sequences. The meta-CATS analysis found 419 of ~29 k base pair positions that had a statistically significant difference between groups (χ^2^ test of independence). This analysis also used a Pearson’s χ^2^ test to determine that 3771 base pair positions significantly differed between one SARS-CoV-2 phylogenetic clade variant (such as omicron) and another SARS-CoV-2 variant. The full results of these analyses are provided in Table S3 and Table S4, respectively.

While meta-CATS performed comparisons of each defined group against all the other groups, the preeminent comparisons were those made against the reference sequence as these better highlighted trends in the observed mutations. Overall, 241 base pair positions achieved statistical significance when compared against the reference sequence (see Table S5). Of these, ~29% of mutations from the reference sequence resulted in a shift toward non bias in any genome, ~20% resulted in a shift toward the SARS-CoV-2 bias, ~12% resulted in a shift toward the HG bias, and ~4% resulted in a shift toward the HPT bias (see Table 1). 

The majority of these significant mutations were located within the Spike (*S*), *orf1a*, and Nucleocapsid (*N*) genes (see Table S5). Across all the statistically significant comparisons to the reference sequence, 21% of comparisons mutated in the first position only, 30% mutated in the second position only, and 25% mutated in the third position only. The remaining 24% of comparisons mutated in a combination of positions (see Table 2); deletions comprised the majority of cases in which all three codon positions were mutated (see Table S5). 

**Figure 3:**
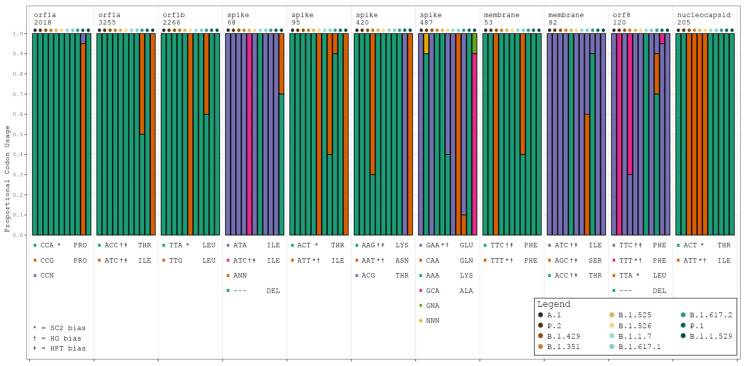
Codon usage trends for all variants at 11 significant codon positions. Stacked barplots display the proportional codon usage for each major variant at each codon position identified as statistically significantly different in both the meta-CATS and HyPhy analyses. The order of bars in each grouping is as follows: A.1 (reference sequence), P.2 (zeta), B.1.429 (epsilon), B.1.351 (beta), B.1.525 (eta), B.1.526 (iota), B.1.1.7 (alpha), B.1.617.1 (kappa), B.1.617.2 (delta), P.1 (gamma), and B.1.1.529 (omicron). Amino acid abbreviations are provided alongside each listed codon. SC2 = SARS-CoV-2. N in any codon denotes an ambiguous base call at the time of sequencing. SARS-CoV-2 codon bias = *, HG codon bias = †, and HPT codon bias = ‡.

HyPhy identified 37 codon sites presenting evidence of statistically significant pervasive selective pressure (see Table S6), of which 13 demonstrated positive selective pressure and 24 demonstrated negative selective pressure. Eleven of the thirty-seven sites also achieved significance in the meta-CATS χ^2^ test of independence (six of the positive pressure sites and five of the negative pressure sites). Codon usage at these 11 sites generally remained consistent with that of the reference sequence (see Figure 3, Table S7). Additionally, and in nearly every case, the designation of the reference sequence’s codon preference (SARS-CoV-2, HG, or HPT) remained unchanged through the course of mutation. These results trend toward those of the Pearson’s χ^2^ test in that the majority of bias shifts were toward either non bias or the baseline SARS-CoV-2 bias. A few exceptions to this were noted, such as codon 420 in the *S* gene, which shifted from a dual HG/HPT bias to a SARS-CoV-2/HG bias in variants B.1.351 and B.1.1.529.

The MISTIC analysis identified substantial evidence of coevolution throughout the viral polyproteins. Two codon sites that were identified as being significant in both the meta-CATS and HyPhy analyses achieved high mutual information (MI) values, which indicates a substantial probability of coevolution (see Table S8). Specifically, codon 120 in *orf8* was linked to codons 16 and 119; while codon 205 in *N* was linked to codons 2, 12, 203, and 204. Each linked codon was itself linked to others, most also carrying high MI values, revealing a network of potentially coevolved amino acid residues throughout the highlighted genes.

## 4. Discussion

The aim of the current work was to determine whether the emerging SARS-CoV-2 variants had undergone mutative pressure in the coding regions of its genome sequence that affected the fitness of the viral population in each phylogenetic variant. To achieve so, we examined the codon bias, significant polymorphisms between variants, and co-evolution within representative genomes from major SARS-CoV-2 variants. With few exceptions, the observed codon bias in the SARS-CoV-2 reference sequence did not agree with the HG or HPT bias. Given that changes to the overall bias observed across all SARS-CoV-2 samples were minute, we conclude that both early and recent strains’ genomes present no evidence of a mutational trajectory influenced by inherent human genomic and pulmonary tissue biases. Rather, the observed mutations appear to occur randomly, and, if shifts in codon usage bias are noted, they reinforce existing biases rather than embrace new ones. However, this may prove to be advantageous to the virus, as its inherent codon bias has been shown to disrupt tRNA pools to the detriment of the host and facilitate evasion of the host’s immune response [18].

We identified eleven codon sites among 121 SARS-CoV-2 samples that present evidence for both statistically significant differences and pervasive positive/negative selective pressure. Positive selection refers to the idea that these sites are conducive to the promotion of diversification, which can potentially lead to fitness advantages which may include evasion of the host’s adaptive immune response. These sites are not critical to the survival of the virus and can, therefore, undergo selection for diversifying traits with no loss of function and, potentially, some gain of function. Negative selection, by contrast, denotes sites that are critical to the function or survival of the virus and cannot be mutated without an accompanying compensatory mutation. Of these sites, six and five demonstrated evidence for positive selection and negative selection, respectively. These findings were further refined by analyzing them for evidence of coevolution, resulting in the identification of *N* codon 205 and *orf8* codon 120. 

Notably, Alonso et al. have found *N* codon region 203–205 to be highly variable and subject to positive selection, which is consistent with our HyPhy result for codon 205 [19]. The nucleocapsid N protein is the most highly expressed of the four SARS-CoV-2 structural proteins. Its purpose is to bind and package the positive-sense RNA within the virion as well as interact with the viral membrane protein during assembly. Herein, codon 205 in *N* is linked to codons 2, 12, 203, and 204. Residues 2 and 12 are in the N-terminal region preceding the well-studied RNA binding domain of protein N. This region is not well conserved in coronaviruses and is, therefore, not part of the CoV-N-NTD superfamily domain [20]. In contrast, residues 203, 204, and 205 lie within a serine-rich subset of the CoV-N-NTD superfamily domain [21]. Further, MISTIC revealed a coevolutionary relationship between codon 205 and codons 203 and 204, which is argued to augment SARS-CoV-2′s capacity for human infection and transmissivity increasing replication, pathogenesis, and fitness in vivo and in vitro [19]. 

The viral orf8 protein is known to have multiple functions. It has been shown to activate adaptive unfolded protein responses, thus suppressing apoptosis, mimic histones to modulate host activity, and directly target the S protein for degradation to avoid the formation of pseudoviruses and decrease the incorporation of S in MHC-1 on the cell’s surface, which could downregulate the host’s immune response [22,23]. Residues 119 and 120 lie within a homodimer interface region, while residue 16 is nearby one (residues 18–24). Additionally, the deletions of codons 119 and 120 documented in the delta variant have been shown to cause structural instability of the orf8 dimer, resulting in a more effective host immune response against the virus due to reduced efficacy in hindering MHC-1 expression [24]. Our result that codon 120 is subject to negative selective pressure validates the designation of *orf8* as being critical to SARS-CoV-2 pathogenicity and suggests that it could be useful as a potential target for future functional, vaccine, and/or therapeutic investigations.

The limitations to this study include the number of variants selected for analysis and the nature of the comparisons. In order to confirm the results presented herein, a more extensive and structured sampling of genomes is suggested in order to create a more representative mutational profile. The study design is limited in that prominent variants are compared against each other and the reference variant. In order to further elucidate whether or not any trends in mutation have arisen, future analyses tracking mutations within developing phylogenetic branches are recommended. Further, the comparisons made in these analyses evaluated the most prominent and, therefore the most successful variants. In order to determine whether the alignment of codon biases with that of human tissues grants some form of evolutionary advantage to the variants in question, it would be prudent to sample a wider range of developing lineages, particularly those which are less successful. Past work has shown that pathogenicity and capacity for replication are strongly dependent on and variable primarily by viral phylogenetic variants [25,26] and that the pathogenicity of individual strains within one variant is more correlated with the demographic, health status, and co-morbidities of the host rather than unique mutations of an individual strain [27]. Elucidatory trends may be uncovered through continuous monitoring of emerging genomic data in the future.

Despite these limitations, we are not aware of any studies that attempted to investigate SARS-CoV-2 variants for evidence of a host-influenced evolutionary trajectory. Future analyses have the potential to corroborate our result that the inherent bias of SARS-CoV-2 is more advantageous than a host-influenced bias, the confirmation of which may influence the discovery of targets for future vaccines and boosters and assist in the development of precautionary measures to slow the spread of the ongoing pandemic. The replication of our results may also validate the bioinformatics pipeline through which the data were processed and serve as a foundation for future analyses seeking to evaluate and select candidates for rigorous wet-lab analyses.

## 5. Conclusions

Overall, these results suggest that prominent variants of SARS-CoV-2 are experiencing pervasive selective pressure at a range of codon sites and indicate that the differences between the codon usage observed at these sites is significantly different from variant to variant. However, these accumulated mutations are insufficient to substantially influence the overall proportional codon usage throughout the genomes of all the tested samples, suggesting that codon usage is stable—and possibly advantageous—despite SARS-CoV-2′s rapid evolution.

## Figures and Tables

**Table 1:** Classification of bias shifts for all significant reference sequence comparisons.

Change Type	N (%)	Number Synonymous (%)	Number Nonsynonymous (%)	Number Deletion (%)
Toward SARS-CoV-2	49/241 (20%)	34/49 (67%)	17/49 (33%)	0/49 (0%)
Toward HG	30/241 (12%)	4/30 (13%)	26/30 (87%)	0/30 (0%)
Toward HPT	10/241 (4%)	7/10 (70%)	3/10 (30%)	0/10 (0%)
Toward non bias	70/241 (29%)	8/70 (11%)	23/70 (33%)	39/70 (56%)

**Table 2:** Number of mutations at each codon position. Displayed is the number and proportion of mutations occurring at a given codon position across 241 codons significantly different from the reference sequence. Categories 1/2, 2/3, and 1/2/3 are independent from all others.

Mutation by Codon Position	N (%)
1	50/241 (21%)
2	71/241 (30%)
3	61/241 (25%)
1/2	6/241 (2%)
2/3	8/241 (3%)
1/2/3	45/241 (19%)

## Data Availability

The data used in these analyses are not shared here as per GISAID policy; however, the accession numbers for all the data are provided in the supplementary materials and may be freely accessed through GISAID.

## Supplementary Materials

The following supporting information can be downloaded at https://www.mdpi.com/article/10.3390/v16020276/s1: Tables S1–S8. Table S1. SARS-CoV-2 GISAID sample metadata; Table S2. Human genome, human pulmonary tissue, and SARS-CoV-2 codon usage; Table S3. Complete meta-CATS χ^2^ test of independence results; Table S4. Complete meta-CATS Pearson’s χ^2^ test results; Table S5. Classification of significant SARS-CoV-2 reference sequence results; Table S6. Complete HyPhy results; Table S7. Codon usage trends at significant codon positions; Table S8. Complete MISTIC analysis results.
